# Predictors of the Quality of Life of University Students: A Cross-Sectional Study

**DOI:** 10.3390/ijerph191912043

**Published:** 2022-09-23

**Authors:** Enrique Ramón-Arbués, Emmanuel Echániz-Serrano, Blanca Martínez-Abadía, Isabel Antón-Solanas, Ana Cobos-Rincón, Iván Santolalla-Arnedo, Raúl Juárez-Vela, Benjamin Adam Jerue

**Affiliations:** 1 Faculty of Health Sciences, Campus Universitario Villanueva de Gállego, Universidad San Jorge, 50830 Saragossa, Spain; 2 H27_20D Transfercult, Investigation Group, Aragón, 50009 Zaragoza, Spain; 3 Faculty of Health Sciences, Zaragoza University, 50009 Zaragoza, Spain; 4 Occupational Health and Prevention Service of the Zaragoza City Council, 50003 Saragossa, Spain; 5 GRUPAC, Department of Nursing, Faculty of Health Sciences, University of La Rioja, 26004 Logroño, Spain; 6 Biomedical Research Center of La Rioja (CIBIR), 26004 Logroño, Spain; 7 Faculty of Communication and Social Sciences, Campus Universitario Villanueva de Gállego, Universidad San Jorge, 50830 Saragossa, Spain

**Keywords:** quality of life, WHOQOL-BREF, universities, young adults, cross-sectional studies

## Abstract

Quality of life (QOL) is a complex and multifaceted concept that has been used to study different aspects of people’s lives, including physical and psychological wellbeing, financial independence, social relationships, personal beliefs and living situation. In this study, we aimed to assess the QOL of a group of Spanish university students and identify associated factors. Method: We completed a cross-sectional study of the QOL of 868 university students using the WHOQOL-BREF questionnaire. In addition, data regarding sociodemographic information and self-esteem (Rosenberg Self-Esteem Scale), physical activity (International Physical Activity Questionnaire—Short version), diet (Spanish Index of Healthy Eating), alcohol consumption (CAGE questionnaire) and sleep quality (Pittsburgh Sleep Quality Index) were collected. Results: A total of 66.2% of participants assessed their QOL positively, while 58.8% favorably evaluated their overall health. Students reported the highest scores for the physical health domain of QOL, whereas they gave the lowest scores for the psychological health domain. Age was inversely associated with QOL. Higher self-esteem and satisfaction with academic performance, as well as sleep and diet quality, were directly associated with higher QOL. The physical health domain of QOL was scored more highly by participants who had a healthy body weight or those who reported moderate levels of physical activity. Higher scores in the social relationships domain of QOL were directly linked to alcohol intake, smoking and low body weight in addition to being inversely associated with screen time. The psychological domain of QOL was lower for those who were overweight or lived alone. Conclusion: Many sociodemographic, academic and behavioral variables are associated with university students’ QOL. The present findings underscore the need to direct further initiatives toward identifying and overcoming barriers to increased QOL for university students.

## 1. Introduction

Quality of life (QOL) is a complex and multifaceted concept that has been used to study different aspects of people’s lives, including physical and psychological wellbeing, financial independence, social relationships, personal beliefs and living situation [1]. The varied contexts in which QOL has been used, however, have made it difficult to define the term in a way that is acceptable to researchers in all fields. Accordingly, starting in the 1960s, researchers proposed a range of definitions of QOL focusing on different factors, including basic human needs, subjective wellbeing, hopes and expectations for the future, and other phenomenological considerations [2]. The World Health Organization (WHO) has defined QOL as “an individual’s perception of their position in life in the context of the culture and value systems in which they live and in relation to their goals, expectations, standards and concerns” [3], and goes on to urge researchers to measure QOL in different cultures and contexts in order to obtain a more holistic view of health and provide better treatment to patients [3].

Adolescence and the early years of adulthood are among the most crucial phases of human development [4]. In these periods of physical, psychological, social and sexual development, adolescents and young adults gradually assume greater responsibility for and autonomy over their own lives; likewise, they develop new attitudes and beliefs about their health and risks to it [5]. For this population, the transition from secondary to higher education can be especially demanding for many reasons, including the pressure to thrive academically, competition between peers, changes in workload and support networks and, on certain occasions, changes in living conditions and being away from family for an extended period of time [6]. The potent mix of biological and social–psychological factors during this period can leave university students especially vulnerable and susceptible to high-risk behavior, whether physical, psychological, or a combination thereof; such behaviors, in turn, can have a negative impact on students’ QOL in both the short and long term [7].

Previous studies have demonstrated how university students, especially those studying health sciences, exhibit lower levels of QOL than the general population [8,9,10]. This body of research has already revealed several factors linked to having a higher QOL among university students, including, but not limited to, the following: satisfaction with their studies [11,12], satisfaction with life [13], being male [14], the absence of signs of depression [15,16] and stress [16,17], being well off economically [12], having healthy sleep habits [18,19] and having a body mass index (BMI) of less than 30 [10,20]. However, there are other factors that have been shown to affect the QOL of the general population, but their influence on the QOL of university students has yet to be properly studied. These factors include diet [21], physical activity [22], smoking [23], alcohol intake [24] and relationship status [25]. Furthermore, most of the scholarly literature on the QOL of university students has focused on specific groups of students: generally those enrolled in health science programs (e.g., medicine, dentistry or nursing). These degree programs are notoriously competitive, give rise to stressful situations, and are punctuated with important rites of passage [26,27]. Given the prevailing focus on this subset of students, there is a dearth of research on the QOL of the general population of university students. In Spain, such research has been limited to only a handful of studies with small sample sizes [28,29,30,31]. The present paper aims to fill in this gap in the literature by studying a broader group of university students from different fields of study and determining which variables are associated with QOL.

## 2. Materials and Methods

### 2.1. Procedure and Participants

**Figure 1:**
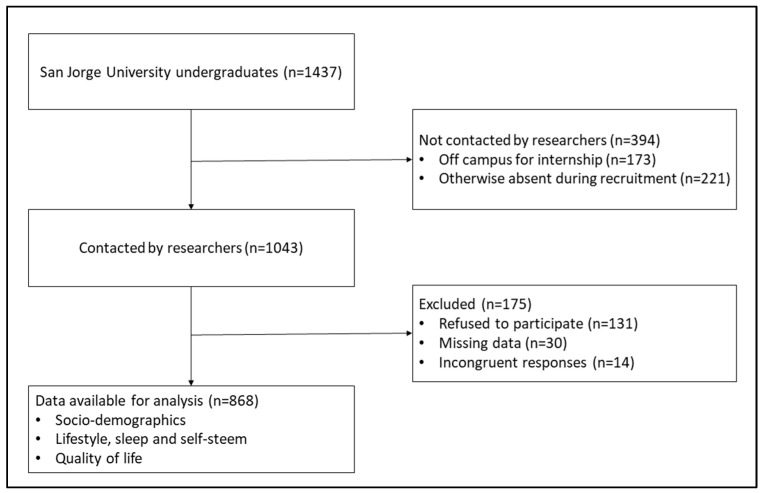
Study flowchart.

A cross-sectional study was carried out among students from three different faculties (Architecture and Technology, Health Sciences and Communication and Social Sciences) at the Universidad San Jorge in Villanueva de Gállego (Aragon, Spain). Both participant recruitment and data collection took place in the classroom during May 2021. A researcher went to various classrooms to explain to students the study’s objectives and provide them with pertinent information about the collection and handling of data. Students were physically provided with explanatory information for participants as well as an informed consent form. Students were assured that all information would be kept confidential. Furthermore, it was explained that students did not have to participate in the study and that they could choose to end their participation at any time. Of the 1437 students enrolled at the university, 912 opted to participate in the study and filled out the questionnaires provided by the researchers. Of the 912 questionnaires that were received, 44 were discarded and not analyzed since they were incomplete or because the provided information was patently untrue (Figure 1).

### 2.2. Data Collection

The questionnaire used to collect data was comprised of two sections, with the first dealing with sociodemographics (e.g., anthropometric, academic and behavioral data) and the second dealing with QOL.

Using a process designed to protect anonymity, participants reported information about their age, gender, studies, place of residence, relationship status, height, weight, smoking habits, use of mobile devices, physical activity, diet, self-esteem, alcohol intake, sleep habits and quality of life.

The short version of the International Physical Activity Questionnaire (IPAQ—Short Version) was used to collect data about physical activity. The short IPAQ asks participants about the intensity, frequency and duration of physical activity carried out over the last seven days. Responses allow each participant’s physical activity to be classified into one of three levels: high, moderate or low [32].

The Spanish Healthy Eating Index (SHEI) was used to assess participants’ diet quality [33]. The Spanish Society for Community Nutrition has designed this research tool, which is an adapted version of the Healthy Eating Index first introduced by Kennedy et al. [34] to better fit the Spanish context. The SHEI consists of 10 items that are scored between 0 and 10. Accordingly, the final score can range between 0 and 100 and is classified as follows: over 80 (healthy diet), between 50 and 80 (diet needing modification) and under 50 (unhealthy). 

Participants’ self-esteem was assessed using the Rosenberg Self-Esteem Scale [35] This questionnaire contains 10 items (with Likert-style options ranging between 1 and 4 points) so that the minimum score is 10 and the maximum is 40. Participants’ self-esteem is then classified using the following levels: 30 and above (high self-esteem), 26–29 (moderate self-esteem) and 25 and below (low self-esteem). Among the Spanish population, this scale has received an internal consistency of 0.87 and a test–retest reliability (within a year) of 0.74 [36].

Data on alcohol intake were gathered by means of the CAGE questionnaire, which has been validated for Spain by Rodríguez Martos et al. [37]. This questionnaire consists of 4 items, each of which has 2 options (“yes” or “no”). Since each affirmative answer is worth 1 point, there is a maximum score of 4 and a minimum of 0. This questionnaire’s sensitivity oscillates between 65 and 100%, and its specificity between 88 and 100% [38].

Participants’ sleep quality was assessed through the Spanish version of the Pittsburgh Sleep Quality Index (PSQI) [39]. This research tool consists of 19 items that comprise 7 subscales. These subscales, each of which is individually scored between 0 (very good) and 3 (very bad), are added together for a final score ranging from 0 to 21. Any score above 5 signals poor sleep quality or pathological difficulties related to sleep. The Spanish version of the PSQI has demonstrated adequate psychometric properties [39,40] and hence has been deemed a useful tool for epidemiological and clinical research. 

The WHOQOL-BREF was used to measure participants’ QOL [41,42]. This questionnaire consists of 26 items, each of which is scored on a 5-point Likert scale (1–5). The first two items assess participants’ overall perception of their QOL (item 1) and health (item 2). The remaining items gather information about four specific domains of QOL: physical health (7 items), psychological health (6 items), social relationships (3 items) and environmental health (8 items). The average score of each domain is used to calculate a raw score for that domain. Following the guidelines provided by the WHO, the sum of the various raw scores can then be converted to a 0–100-point scale, with higher scores denoting a higher QOL [41]. The WHOQOL-BREF has been repeatedly used to study university students’ QOL [14,43,44] and the Spanish version has demonstrated positive psychometric properties during its validation for a clinical population [45]. 

### 2.3. Data Analysis

The Kolmogorov–Smirnov test and graphic analysis were used to check the normality of the data distribution. The averages and standard deviations are given to present the results of the descriptive analysis of each WHOQOL-BREF domain. Cronbach’s alpha was used to assess the reliability of the Spanish version of the WHOQOL-BREF for the sample, with internal consistency being considered good when values were equal to or higher than 0.7. The Pearson correlation coefficient (PCC) was used to determine the correlation between the different WHOQOL-BREF domains. The bivariate relationships between different variables and QOL scores were analyzed using the Pearson or Spearman correlation coefficient, a Student’s *t*-test and ANOVA.

Different multiple linear regression models (stepwise method using a probability of F to enter ≤ 0.05 and to exit ≤ 0.10) were carried out in order to identify independent predictors of QOL. Age and CAGE score variables showed a significant amount of asymmetry and were log-transformed for this analysis. Furthermore, a collinearity analysis was used to discard from the regression models any factor that presented a tolerance value or variance inflation factor (VIF) close to 1 and a condition index less than 30. Data codification, processing and analysis were completed using the statistical software Statistical Package for the Social Sciences (SPSS version 21 for Windows, IBM Corp., Chicago, IL, USA), accepting a level of significance of *p* < 0.05.

## 3. Results

### 3.1. Demographic Characteristics 

A total of 868 university students participated in this study. The average age was 22.84 ± 7.51 and most participants were female (78.2%). Most students were enrolled in a health science program (61.3%), had a healthy body weight (77.4%), lived at home with parents/family (69.4%), were not smokers (67.7%) and had a diet that was either unhealthy or needed modification (82.2%). Table 1 provides further information about the demographics and habits of the sample. 

### 3.2. WHOQOL-BREF scores and their reliability

**Figure 2:**
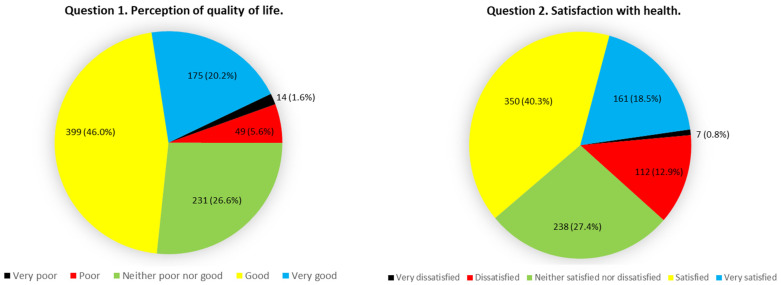
Participants’ perception of their quality of life and satisfaction with their health.

The QOL scores in all domains showed a normal distribution. A total of 66.2% of participants positively evaluated their QOL, while 58.8% were satisfied with their overall health (WHOQOL-BREF items 1 and 2) (Figure 2). 

The following scores were reported for items 1 and 2, as well as the four QOL domains: perception of QOL (3.77 ± 0.88), overall health (3.63 ± 0.95), physical health (76.08 ± 14.17), psychological health (65.92 ± 15.79), social relationships (72.55 ± 19.83) and environmental health (73.77 ± 13.37). The reliability analysis gave Cronbach alpha values between 0.71 and 0.979 for the various domains (Table 2). Nevertheless, it is worth mentioning that since the third domain (social relationships) only consists of three items, the results should be viewed with caution. 

Statistically significant bivariate correlations were found between the four domains of QOL. There were also statistically significant correlations between general perception of QOL and overall health and the scores obtained in the four domains. The strength of the correlation between the different domains ranged from moderate to strong (Pearson’s r between 0.302 and 0.553) (Table 3). 

### 3.3. The Relationship between Sociodemographic Variables and QOL: Multivariant Analysis

The physical health domain was directly associated with satisfaction with academic performance and increased screen time, while it was inversely related to low body weight, obesity, increased alcohol intake (assessed with the CAGE questionnaire) and high levels of physical activity. Being older, female, overweight and having an increased alcohol intake were all associated with lower QOL in the psychological health domain. Satisfaction with school, low body weight, low levels of physical activity, being a smoker, increased alcohol intake and having a stable partner all correlated with higher levels of satisfaction in the social relationships domain, whereas being older and having more screen were associated with lower levels of satisfaction in the same domain. There was a direct relationship between the environmental health domain and academic satisfaction, increased screen time, healthy body weight, being younger and having lower levels of alcohol consumption. It is also worth noting that participants in the final years of their university studies and those that had flat mates reported lower scores in the environmental health domain. Furthermore, self-esteem and sleep quality were associated with a higher QOL in all domains, and healthier diets were linked to stronger scores in the social relationships and environmental health domains. The goodness-of-fit of the different regression models ranged between r^2^ = 0.351 and r^2^ = 0.595 (Table 4).

## 4. Discussion

The present study has sought to assess the QOL of Spanish university students and identify associated predictive factors. A total of 66.2% of the study participants assessed their QOL favorably, while 58.8% positively rated their overall health. Notably, these numbers are lower than those reported in the National Health Survey for the general Spanish population [46]. The participants’ responses concerning their overall perception of QOL and overall health gave average values of 3.77 ± 0.88 and 3.63 ± 0.95, respectively (minimum 1 and maximum 5). These findings are analogous to those reported in other studies of university students from Spain [47], Europe [47,48] and around the world [14,49].

In the present sample, physical and psychological health were the highest and lowest scoring domains, respectively (76.08 ± 14.17 and 65.92 ± 15.79). While the body of published literature on the QOL of university students has been inconclusive concerning which domain university students score the highest, there is a recurring pattern that psychological health receives the lowest score [47,50,51]. It is likely that the differences that emerge in the literature over the strongest QOL domain can be explained in terms of contextual, cultural and socio-economic differences between different countries, communities and cultures; more specifically, we should be aware that there are meaningful differences in the expectations and customs of university students from one context to the next and that such differences surely exercise an influence over QOL.

Previous research has suggested that the first [52] and last [53] years of university are especially punctuated with stressful situations, a fact that could lead to changes in QOL from one year to the next. In our sample, however, age (and not year of study) was the variable that was inversely associated with the psychological, environmental and social domains of QOL. Such a finding could be explained by the particular characteristics of the university from which participants were recruited, since they can study remotely if there are conflicts in their work and school schedules. Given that students who work tend to be older, it would be reasonable that the psychological health, social relationships and environmental health of older students would be negatively impacted due to a greater number of responsibilities (i.e., balancing school and work at once). 

Being female has repeatedly been associated with a lower QOL for university students [10,12,54]. This finding has often been explained in the following terms: stress is negatively associated with QOL [17,55] and women tend to identify a greater range of situations as stressful, and are more negatively impacted by stressful situations than men [50]. In the present study, however, gender was not a significant predictor for QOL. Likewise, QOL was not linked to a student’s field of study. These results diverge from most previously reported findings which have suggested that studying health sciences is associated with higher levels of stress and worse QOL due to a range of factors, such as heavy workloads, exacting standards, and the need to work with real-life patients during clinical internships [9,56].

Various studies have argued for the beneficial impact of a balanced diet on QOL, as well as the physical and mental health of the general population [57,58], and university students in particular [59,60]. In this regard, diet quality was only linked to higher QOL in the domains of social relationships and environmental health, and not to the physical or psychological health domains. The direct link between physical activity and higher QOL is plausible from a biological perspective, and has been observed in previous studies [61,62]. However, the present analysis of this association provided surprising results since it revealed a U-shaped relationship for the domains of psychological and environmental health: participants with either high or low levels of physical activity reported a higher QOL. Perhaps these results can be partially explained by the questionnaire used to measure physical activity (IPAQ—Short Version), since it does not draw potentially meaningful distinctions between competitive/recreational activities, individual/team activities or indoor/outdoor activities. All these factors could plausibly influence the QOL domains of psychological health, social relationships and environmental health. Furthermore, the IPAQ—Short Version relies on participants’ ability to recall what exercise they had undertaken over the last seven days. Accordingly, it would be of particular interest to measure levels of physical activity more objectively in future studies on the QOL of Spanish university students.

For the participants of the present study, there was a direct correlation between self-esteem and QOL in all domains. This relationship has previously been reported for university students [63]. Self-esteem is a multidimensional concept that is influenced by a large range of factors, such as self-image, perception of social support, life experience or the achievement of goals. Thus, a high level of self-esteem improves one’s mindset, positively influences health-related behaviors and fosters emotional wellbeing and stability [64]. Inversely, low levels of self-esteem are correlated with worse mental health [65,66] and ultimately lower QOL. As previous studies have found [67,68], higher levels of satisfaction with academic performance were linked to higher scores in the physical and psychological health domains of QOL. One possible explanation for the relationship between academic success and higher QOL in the physical health domain could be that students with higher energy levels are able to spend more time studying, which is then reflected in higher grades [69]. In a similar vein, different studies have shown how physical activity can improve young people’s academic habits and skills and hence boost their academic performance [70,71]. As far as the psychological health domain is concerned, fulfilling one’s own academic goals seems to lead to increased efficiency and self-esteem, both of which would positively impact QOL in this domain [11].

In this study, the variable that was most strongly correlated to QOL (in all domains) was sleep quality, an association that has repeatedly been reported in the literature [18,61]. University students may have trouble sleeping due to physiological factors and, especially, behavioral ones, such as poor sleep habits, the use of stimulants, alcohol intake, the consumption of caffeinated or energy drinks or an overuse of screens [72,73]. Furthermore, poor sleep quality is related to stress [74], mood swings [75,76] and lower academic achievement [77,78], all of which could lead to diminishing QOL.

Due to their health implications, several of the present study’s findings deserve special attention: being a smoker, consuming alcohol and having a low body weight (BMI < 18.5 kg/m^2^) were all related to higher scores in the social relationships domain of QOL. It must be acknowledged that in many western societies, beauty is associated with being thin, and both alcohol and tobacco are widely considered to be important for socializing. Today, there are still prevalent attitudes that encourage insufficient diet as well as normalize drinking and smoking, while downplaying their health consequences [79,80]. Public health authorities should be engaged in forging a broad social consensus that acknowledges the importance of a healthy diet and dissuades adolescents and young adults from taking up smoking and consuming alcohol on a regular basis. On its own, information campaigns will not be sufficient to address the problem since this action needs to be buttressed by other effective policies (e.g., stricter regulations on advertising as well as the tobacco and alcohol industries generally) to protect the health of young people.

While the vast majority of published literature on the QOL of university students has focused on those enrolled in specific degree programs, the present study is, as far as the authors are aware, the first to investigate the QOL of the general population of Spanish university students and a broad range of sociodemographic and behavioral variables. When combined with the large sample size, the approved techniques for collecting data and the plausibility of the results, the scope and design of the study have led the authors to posit that the results can be taken as representative of the larger body of Spanish university students. Accordingly, the authors believe that the present portrait is reliable and can serve as a point of departure for developing and implementing new interventions aimed at improving the various domains of QOL for Spanish university students. Despite this confidence, several limitations of the present study must be acknowledged. First, the transversal design has made it possible to detect associations, but it does not allow us to determine a cause-and-effect relationship or the direction of influence; this is also true for potentially bidirectional relationships, such as the link between QOL and alcohol and tobacco intake or sleep quality. Second, given that the findings come from only one academic center, as a limitation we emphasize that it is not possible to extrapolate them to the total number of centers in our country. Third, our data were collected when the COVID-19 pandemic and the resulting public health measures were well underway in Spain. This fact provides valuable information concerning the QOL of Spanish university students in that specific context and, in all likelihood, the results reflect the pandemic’s impact on this population; however, these results do not allow us to determine the precise way in which the pandemic has influenced university students’ QOL. Fourth, our data were collected outside of an exam period, which means that results could very well have been different (i.e., lower scores in the psychological and environmental health domains) had students completed the study questionnaire during an exam period. This hypothesis is based on the assumption that some students see exams as high-stakes events that are emotionally taxing and can increase levels of stress [81,82]. As a result, QOL could fluctuate throughout the academic year, reaching a low point during exams.

Finally, it is quite possible that our theoretical model could be further refined through the inclusion of even more variables, such as satisfaction with one’s field of study, the institutional support available to students (in the classroom and through more general guidance counseling) or the techniques and resources that students use to overcome challenges. Future studies in this area ought to address the limitations of the present study. Accordingly, it would be desirable to conduct longitudinal studies and include an increased number of variables related to education and study habits.

## 5. Conclusions

Spanish university students reported medium–high levels of QOL (3.77 out of 5), with the physical health domain scoring the highest and the psychological one the lowest. These results, however, showed lower levels of perceived QOL among university students than the general population. A suite of sociodemographic (e.g., age and BMI), academic (satisfaction with academic performance), emotional (self-esteem) and behavioral (the consumption of alcohol and tobacco, sleep practices and diet) factors have been identified to help us significantly predict the QOL of this population.

The present results provide relevant information for policymakers hoping to implement strategies to identify barriers in the physical, psychological, social and environmental domains in order to increase the overall QOL of university students. In this vein, putting into practice activities geared towards improving physical health and the level of socialization, designing health education programs meant to modify unhealthy lifestyle choices and training educators to identify the immediate psychological needs of the student body would all be effective and impactful measures to improve the QOL of university students.

## Figures and Tables

**Table 1:** Participant characteristics and quality of life.

		Physical Health Domain	Psychological Health Domain	Social Relationships Domain	Environmental Health Domain
	Number (%) / Average ± SD	Average ± SD / Correlation Coefficient	Average ± SD / Correlation Coefficient	Average ± SD / Correlation Coefficient	Average ± SD / Correlation Coefficient
Age	22.84 ± 7.51	−0.112 *	−0.084 **	−0.014	−0.138 *
Under 21	525 (60.5%)	77.48 ± 13.15	67.09 ± 16.18	74.29 ± 19.00	75.40 ± 13.77
Between 21 and 29	231 (26.6%)	72.82 ± 14.37	6315 ± 14.97	70.21 ± 18.09	70.73 ± 12.54
30 or older	112 (12.9%)	76.25 ± 17.13 *	66.13 ± 15.03 *	69.19 ± 25.53 *	72.44 ± 11.98 *
Degree in health sciences	532 (61.3%)	75.77 ± 12.90	65.83 ± 15.33	73.55 ± 19.57	73.65 ± 13.66
Other degrees	336 (38.7%)	76.57 ± 15.99	66.07 ± 16.52	70.96 ± 20.16	73.97 ± 12.92
First-year students	456 (52.5%)	76.071 ± 3.73	67.79 ± 13.90	74.32 ± 20.36	74.85 ± 12.46
Other years	412 (47.5%)	76.09 ± 14.67	63.85 ± 17.45 *	70.59 ± 19.06 *	72.58 ± 14.24 **
Male	189 (21.8%)	80.41 ± 13.55	71.11 ± 12.77	73.41 ± 19.82	77.44 ± 13.76
Female	679 (78.2%)	74.88 ± 14.12 *	64.47 ± 16.26 *	72.31 ± 19.84	72.75 ± 13.10 *
BMI	22.20 ± 3.18	0.032	−0.052	−0.191 *	−0.077 **
Low body weight (<18.5)	84 (9.7%)	70.42 ± 17.09	65.67 ± 20.59	77.58 ± 16.03	70.00 ± 14.66
Healthy body weight (18.5–24.9)	672 (77.4%)	77.40 ± 13.67	67.23 ± 14.95	73.00 ± 19.38	75.40 ± 12.98
Overweight (25–29.9)	70 (8.1%)	73.30 ± 12.13	56.90 ± 12.22	64.40 ± 18.73	66.30 ± 13.16
obese (≥30)	42 (4.8%)	71.00 ± 14.81 *	60.50 ± 17.78	68.83 ± 29.56	67.83 ± 9.85 *
Lives alone	42 (4.8%)	84.50 ± 14.19	61.67 ± 13.75	72.00 ± 27.42	76.00 ± 15.19
Lives with flat mates	224 (25.8%)	73.16 ± 14.69	69.25 ± 15.52	73.81 ± 22.19	72.19 ± 13.18
Lives with parents/family members	602 (69.4%)	76.58 ± 13.70 *	64.98 ± 15.86 *	72.12 ± 18.24	74.21 ± 13.28
Single	476 (54.8%)	75.43 ± 14.38	64.15 ± 16.63	67.78 ± 19.76	72.81 ± 14.40
In a stable relationship	392 (45.2%)	76.88 ± 13.89	68.07 ± 14.47 *	78.34 ± 18.34 *	74.95 ± 11.93 **
Satisfaction with academic performance (min. 1–max. 5)	3.35 ± 0.86	0.140 *	0.243 *	0.033	0.078 **
Low levels of physical activity	231 (26.6%)	74.33 ± 15.94	64.70 ± 15.90	74.27 ± 21.53	73.82 ± 14.58
Moderate levels of physical activity	371 (42.7%)	77.81 ± 13.74	65.77 ± 15.86	72.43 ± 18.72	71.62 ± 13.55
High levels of physical activity	266 (30.6%)	75.18 ± 12.86 *	67.18 ± 15.59	71.21 ± 19.76	76.74 ± 11.38 *
Screen time (not work/school related)	3.40 ± 1.72	0.055	−0.005	0.037	0.062
Screen time < 3 h per day	329 (37.9%)	76.28 ± 15.96	65.91 ± 16.66	70.34 ± 19.92	73.66 ± 12.83
Screen time ≥ 3 h per day	539 (62.1%)	75.96 ± 12.97	65.92 ± 15.27	73.90 ± 19.67 **	73.84 ± 13.71
Nonsmoker	588 (67.7%)	76.54 ± 14.97	66.36 ± 16.85	70.05 ± 19.50	73.56 ± 13.61
Smoker	280 (32.3%)	75.13 ± 12.32	65.00 ± 13.30	77.80 ± 19.53 *	74.23 ± 12.89
CAGE score	0.62 ± 0.91	−0.042	−0.098 *	0.073 **	−0.024
Responsible alcohol consumption (CAGE < 2)	742 (85.5%)	76.41 ± 14.24	66.48 ± 16.43	71.90 ± 20.46	73.96 ± 13.41
Risky alcohol consumption (CAGE ≥ 2)	126 (14.5%)	74.17 ± 13.68	62.61 ± 10.86 *	76.39 ± 15.15 *	72.67 ± 13.18
Rosenberg scale score	31.48 ± 5.93	0.489 *	0.682 *	0.494 *	0.471 *
High self-esteem	560 (64.5%)	80.84 ± 12.12	73.14 ± 10.73	77.69 ± 18.43	77.63 ± 12.40
Moderate self-esteem	168 (19.4%)	68.83 ± 11.31	58.21 ± 12.31	66.42 ± 17.73	67.04 ± 10.78
Low self-esteem	140 (16.1%)	65.75 ± 15.70 *	46.30 ± 15.57 *	59.35 ± 19.54 *	66.45 ± 13.78 *
Pittsburgh Sleep Quality Index score	6.39 ± 3.58	−0.555 *	−0.519 *	−0.310 *	−0.332 *
Without sleep problems	420 (48.4%)	82.37 ± 11.85	73.50 ± 12.25	78.40 ± 18.62	79.10 ± 13.09
Poor sleep quality	448 (51.6%)	70.19 ± 13.64 *	58.81 ± 15.45 *	67.06 ± 19.38 *	68.78 ± 11.61 *
SHEI score	69.88 ± 11.29	−0.051	0.116 *	0.037	0.232 *
Unhealthy diet	49 (5.6%)	70.43 ± 16.79	60.86 ± 17.08	63.43 ± 23.13	64.29 ± 10.01
Diet needing changes	665 (76.6%)	76.98 ± 14.73	65.51 ± 16.39	73.17 ± 20.11	73.36 ± 13.32
Healthy diet	154 (17.7%)	74.00 ± 9.50 *	69.32 ± 11.67 *	72.77 ± 16.66 *	78.59 ± 12.65 *

* *p* < 0.01, ** *p* < 0.05.

**Table 2:** Participant scores on the WHOQOL-BREF and reliability for each domain.

	Average ± SD	Minimum and Maximum Scores Possible	Minimum and Maximum Scores Obtained	Cronbach’s Alpha
Item 1. Perception of QOL	3.77 ± 0.88	1–5	1–5	-----
Item 2. Overall health	3.63 ± 0.95	1–5	1–5	-----
Physical health domain	76.08 ± 14.17	0–100	31–100	0.71
Psychological health domain	65.92 ± 15.79	0–100	6–94	0.79
Social relationship domain	72.55 ± 19.83	0–100	19–100	0.76
Environmental health domain	73.77 ± 13.37	0–100	44–100	0.72

**Table 3:** Bivariate correlations between different domains of the WHOQOL-BREF.

	Item 1: Perceived QOL	Item 2: Overall Health	Physical Health Domain	Psychological Health Domain	Social Relationships Domain
Item 1: Perception of QOL	-----	0.539 *	-----	-----	-----
Physical health domain	0.468 *	0.421 *	-----	-----	-----
Psychological health domain	0.472 *	0.377 *	0.553 *	-----	-----
Social relationships domain	0.302 *	0.128 *	0.312 *	0.542 *	-----
Environmental health domain	0.478 *	0.315 *	0.502 *	0.538 *	0.419 *

* Correlations are significant at the level of 0.01 (bilateral).

**Table 4:** Multivariate analysis of factors associated with the different QOL domains.

	Physical Health Domain B (95% CI)	Psychological Health Domain B (95% CI)	Social Relationships Domain B (95% CI)	Environmental Health Domain B (95% CI)
Age (years) (log)	-----	−0.120 (−0.219, −0.020) **	−0.477 (−0.641, −0.313) *	−0.151 (−0.271, −0.031) **
Year of study (Ref. first year)				
Other years	-----	-----	-----	−2.049 (−3.918, −0.180) **
Gender (Ref. Male)				
Female	-----	−1.493 (−3.227, 0.241)	-----	-----
BMI (Ref. healthy body weight, 18.5–24.9 kg/m^2^)				
Low body weight (<18.5)	−6.051 (−8.810, −3.291) *	3.044 (0.641, 5.447) **	5.792 (2.037, 9.547)*	−2.976 (−5.610, −0.343) **
Overweight (25–29.9)	-----	−4.049 (−6.704, −1.394) *	-----	−3.233 (−6.118, −0.348) **
Obese (≥30)	−6.159 (−9.801, −2.517) *	−4.297 (−7.645, −0.950) **	-----	−5.227 (−8.868, −1.585) **
Living situation (Ref. lives alone)				
Lives with flat mates	−8.341 (−12.238, −4.444) *	9.887 (6.361, 13.414) *	-----	−2.800 (−4.914, −0.685) *
Lives with parents/family	−3.407 (−7.164, 0.350)	6.808 (3.370, 10.246) *	-----	-----
Relationship status (Ref. single)				
In a stable relationship	-----	-----	9.904 (7.678, 12.130) *	1.869 (0.300, 3.437) **
Satisfaction with academic performance	1.189 (0.142, 2.236) **	2.355 (1.508, 3.202) *	----	-----
Physical activity (Ref. moderate physical activity)				
Low physical activity	-----	2.450 (0.715, 4.185) *	4.586 (2.112, 7.060) *	4.172 (2.326, 6.019) *
High physical activity	−2.057 (−3.830, −0.284) **	1.852 (0.160, 3.544) **	-----	4.918 (2.995, 6.841) *
Screen time (hours)	0.579 (0.090, 1.069) **	-----	−0.641 (−1.392, 0.110)	0.706 (0.172, 1.241) **
Smoking (Ref. Nonsmoker)				
Smoker	-----	-----	5.256 (2.779, 7.733) *	-----
CAGE score (log)	−1.688 (−2.573, −0.802) *	−0.916 (−1.679, −0.153) **	1.770 (0.570, 2.970) *	−0.753 (−1.593, 0.086)
Rosenberg scale score	0.846 (0.705, 0.987) *	1.543 (1.417, 1.668) *	1.457 (1.261, 1.652) *	0.773 (0.637, 0.908) *
Sleep (Ref. without sleep problems)				
Poor sleep quality	−7.600 (−9.261, −5.940) *	−8.032 (−9.509, −6.555) *	−6.290 (−8.607, −3.972) *	−6.476 (−8.062, −4.889) *
SHEI score	-----	-----	0.115 (0.018, 0.212) **	0.105 (0.037, 0.173) *
R^2^ (R^2^ corrected)	0.359 (0.351)	0.601 (0.595)	0.385 (0.377)	0.350 (0.339)

Age and CAGE score are expressed as log-transformed. Does not enter in the model; * *p* < 0.01; ** *p* < 0.05.

## Data Availability

On request to the first author.

## References

[1] Karimi M., Brazier J. (2016). Health, Health-Related Quality of Life, and Quality of Life: What is the Difference?. PharmacoEconomics.

[2] Moons P., Budts W., De Geest S. (2006). Critique on the conceptualisation of quality of life: A review and evaluation of different conceptual approaches. Int. J. Nurs. Stud..

[3] The WHOQOL Group (1995). The World Health Organization Quality of Life assessment (WHOQOL):Position paper from the World Health Organization. Soc. Sci. Med..

[4] Faílde Garrido J.M., Ruiz Soriano L., Pérez Fernández M.R., Lameiras Fernández M., Rodríguez Castro Y. (2019). Evolution of quality of life and health-related behaviors among Spanish university students. Int. J. Health Plan. Manag..

[5] Gray K.M., Squeglia L.M. (2018). Research Review: What have we learned about adolescent substance use?. J. Child Psychol. Psychiatry Allied Discip..

[6] Ramón-Arbués E., Abadía B.M., López J.M.G., Serrano E.E., García B.P., Vela R.J., Portillo S.G., Guinoa M.S. (2019). Eating behavior and relationships with stress, anxiety, depression and insomnia in university students. Nutr. Hosp..

[7] Nur N., Kıbık A., Kılıç E., Sümer H. (2017). Health-related Quality of Life and Associated Factors Among Undergraduate University Students. Oman Med. J..

[8] Ghassab-Abdollahi N., Shakouri S.K., Aghdam A.T., Farshbaf-Khalili A., Abdolalipour S., Farshbaf-Khalili A. (2020). Association of quality of life with physical activity, depression, and demographic characteristics and its predictors among medical students. J. Educ. Health Promot..

[9] Messina G., Quercioli C., Troiano G., Russo C., Barbini E., Nisticò F., Nante N. (2016). Italian medical students quality of life: Years 2005–2015. Ann. Di Ig. Med. Prev. E Di Comunita.

[10] Serinolli M.I., Novaretti M.C.Z. (2017). A cross-sectional study of sociodemographic factors and their influence on quality of life in medical students at Sao Paulo, Brazil. PLoS ONE.

[11] Shareef M.A., AlAmodi A.A., Al-Khateeb A.A., Abudan Z., Alkhani M.A., Zebian S.I., Qannita A.S., Tabrizi M.J. (2015). The interplay between academic performance and quality of life among preclinical students. BMC Med. Educ..

[12] Solis A.C., Lotufo-Neto F. (2019). Predictors of quality of life in Brazilian medical students: A systematic review and meta-analysis. Braz. J. Psychiatry.

[13] Yildirim Y., Kilic S.P., Akyol A.D. (2013). Relationship between life satisfaction and quality of life in Turkish nursing school students. Nurs. Health Sci..

[14] Al-Shibani N., Al-Kattan R. (2019). Evaluation of quality of life among dental students using WHOQOL-BREF questionnaire in Saudi Arabia: A cross sectional study. Pak. J. Med. Sci..

[15] Pillay N., Ramlall S., Burns J.K. (2019). Spirituality, depression and quality of life in medical students in KwaZulu-Natal. South Afr. J. Psychiatry SAJP J. Soc. Psychiatr. South Afr..

[16] Seo E.J., Ahn J.-A., Hayman L.L., Kim C.-J. (2018). The Association Between Perceived Stress and Quality of Life in University Students: The Parallel Mediating Role of Depressive Symptoms and Health-Promoting Behaviors. Asian Nurs. Res..

[17] Labrague L.J., McEnroe-Petitte D.M., Papathanasiou I.V., Edet O., Tsaras K., Christos K.F., Fradelos E.C., Rosales R.A., Cruz J., Leocadio M. (2018). A cross-country comparative study on stress and quality of life in nursing students. Perspect. Psychiatr. Care.

[18] Kwon S.J., Kim Y., Kwak Y. (2020). Relationship of sleep quality and attention deficit hyperactivity disorder symptoms with quality of life in college students. J. Am. Coll. Health.

[19] Rezaei O., Mokhayeri Y., Haroni J., Rastani M.J., Sayadnasiri M., Ghisvand H., Noroozi M., Armoon B. (2017). Association between sleep quality and quality of life among students: A cross sectional study. Int. J. Adolesc. Med. Health.

[20] Wanden-Berghe C., Martín-Rodero H., Rodríguez-Martín A., Ruiz J.P.N., De Victoria E.M., Sanz-Valero J., García-González Á., Vila A., Alonso M.V., Marí J.A.T. (2014). Quality of life and its determinants in Spanish university students of health sciences factors. Nutr. Hosp..

[21] Feasel-Aklilu S., Marcus A., Parrott J.S., Peters E., Byham-Gray L. (2018). Is Nutrition Specific Quality of Life Associated With Nutritional Status?. J. Ren. Nutr..

[22] Kanesarajah J., Waller M., Whitty J.A., Mishra G.D. (2018). Physical activity and body mass shape quality of life trajectories in mid-age women. Aust. N. Z. J. Public Health.

[23] Goldenberg M., Danovitch I., IsHak W.W. (2014). Quality of life and smoking. Am. J. Addict..

[24] Santos M.V.F.D., Campos M.R., Fortes S.L.C.L. (2019). Relationship of alcohol consumption and mental disorders common with the quality of life of patients in primary health care. Cienc. Saude Coletiva.

[25] Verma A.K., Schulte P.J., Bittner V., Keteyian S.J., Fleg J.L., Piña I.L., Swank A.M., Fitz-Gerald M., Ellis S.J., Kraus W.E. (2017). Socioeconomic and partner status in chronic heart failure: Relationship to exercise capacity, quality of life, and clinical outcomes. Am. Heart J..

[26] Almojali A.I., Almalki S.A., Alothman A.S., Masuadi E.M., Alaqeel M.K. (2017). The prevalence and association of stress with sleep quality among medical students. J. Epidemiol. Glob. Health.

[27] Pulido-Martos M., Augusto-Landa J.M., Lopez-Zafra E. (2012). Sources of stress in nursing students: A systematic review of quantitative studies. Int. Nurs. Rev..

[28] Fernández-Martínez E., Onieva-Zafra M.D., Parra-Fernández M.L. (2019). The Impact of Dysmenorrhea on Quality of Life Among Spanish Female University Students. Int. J. Environ. Res. Public Health.

[29] Franquelo-Morales P., Sánchez-López M., Notario-Pacheco B., Miota-Ibarra J., Lahoz-García N., Gómez-Marcos M.Á., Martínez-Vizcaíno V. (2018). Association Between Health-Related Quality of Life, Obesity, Fitness, and Sleep Quality in Young Adults: The Cuenca Adult Study. Behav. Sleep Med..

[30] Latorre-Román P.Á., Gallego-Rodríguez M., Mejía-Meza J.A., García-Pinillos F. (2015). Alcohol, and tobacco consumption and sports practice in Mexican and Spanish university students and the association between quality of life and health and sensation seeking. Gac. Med. De Mex..

[31] Martín-Espinosa N.M., Garrido-Miguel M., Martínez-Vizcaíno V., González-García A., Redondo-Tébar A., Cobo-Cuenca A.I. (2020). The Mediating and Moderating Effects of Physical Fitness of the Relationship between Adherence to the Mediterranean Diet and Health-Related Quality of Life in University Students. Nutrients.

[32] The International Physical Activity Questionnaire Group The International Physical Activity Questionnaire Group Guidelines for Data Processing and Analysis of the International Physical Activity Questionnaire. http://www.ipaq.ki.se/scoring.pdf.

[33] Norte Navarro A.I., Ortiz Moncada R. (2011). Spanish diet quality according to the healthy eating index. Nutr. Hosp..

[34] Kennedy E.T., Ohls J., Carlson S., Fleming K. (1995). The Healthy Eating Index: Design and applications. J. Am. Diet. Assoc..

[35] Rosenberg M. (1995). Society and the Adolescent Self-Image.

[36] Vázquez-Morejón A., Jiménez-García R., Vázquez-Morejón R. (2004). Escala de autoestima de Rosenberg. Fiabilidad y validez en población clínica española. Apunt. De Psicol..

[37] Rodríguez Martos A., Navarro R., Vecino C., Pérez R. (1986). Validación de los cuestionarios KFA (CBA) y CAGE para el diagnóstico del alcoholismo. Drog. -Alcohol..

[38] Morales-Rueda A., Rubio-Valladolid G. (1997). Diagnóstico y tratamiento de los problemas relacionados con el alcohol en atención primaria. MEDIFAM.

[39] Royuela-Rico A., Macías-Fernández J. (1997). Propiedades clinimétricas de la versión castellana del cuestionario de Pittsburgh. Vigilia-Sueño.

[40] Jiménez-Genchi A., Monteverde-Maldonado E., Nenclares-Portocarrero A., Esquivel-Adame G., de la Vega-Pacheco A. (2008). Reliability and factorial analysis of the Spanish version of the Pittsburg Sleep Quality Index among psychiatric patients. Gac. Med. De Mex..

[41] Skevington S.M., Lotfy M., O’Connell K.A., WHOQOL Group (2004). The World Health Organization’s WHOQOL-BREF quality of life assessment: Psychometric properties and results of the international field trial. A report from the WHOQOL group. Qual. Life Res..

[42] World Health Organization, Division of Mental Health (1996). WHOQOL-BREF: Introduction, Administration, Scoring and Generic Version of the Assessment: Field trial Version, December 1996. (No. WHOQOL-BREF). World Health Organization..

[43] Ridner S.L., Keith R.J., Walker K.L., Hart J.L., Newton K.S., Crawford T.N. (2018). Differences in quality of life among college student electronic cigarette users. AIMS Public Health.

[44] Victor F.F., Souza A.I., Barreiros C.D.T., de Barros J.L.N., da Silva F.A.C., Ferreira A.L.C.G. (2019). Quality of Life among University Students with Premenstrual Syndrome. Rev. Bras. De Ginecol. E Obstet. Rev. Da Fed. Bras. Das Soc. De Ginecol. E Obstet..

[45] Pedrero-Pérez E.J., Barreda-Marina M.A., Bartolomé-Gil C., Bosque-Coro S., Callejo-Escobar J., Ema-López I., Dominguez-Aranda M.A., Ferrero-Herreros Y.E., Galera-García Ó., Garrido-Ureña B. (2018). Quality of life in patients treated with methadone: The WHOQOL-BREF, psychometric study and application results. An. De Psicol..

[46] Ministerio de Sanidad Encuesta Nacional de Salud de España (ENSE). Portal Estadístico Área de Inteligencia de Gestión..

[47] Kupcewicz E., Grochans E., Kadučáková H., Mikla M., Jóźwik M. (2020). Analysis of the Relationship between Stress Intensity and Coping Strategy and the Quality of Life of Nursing Students in Poland, Spain and Slovakia. Int. J. Environ. Res. Public Health.

[48] Ilić I., Šipetić S., Grujičić J., Mačužić I.Ž., Kocić S., Ilić M. (2019). Psychometric Properties of the World Health Organization’s Quality of Life (WHOQOL-BREF) Questionnaire in Medical Students. Medicina.

[49] Irribarra T.L., Mery I.P., Lira S.M.J., Campos D.M., González L.F., Irarrázaval D.S. (2018). Quality of life scores among 411 medical students. Rev. Med. De Chile.

[50] Backhaus I., D’Egidio V., Saulle R., Masala D., Firenze A., De Vito E., Mannocci A., La Torre G. (2020). Health-related quality of life and its associated factors: Results of a multi-center cross-sectional study among university students. J. Public Health.

[51] Cruz J.P., Felicilda-Reynaldo R.F.D., Lam S.C., Contreras F.A.M., Cecily H.S.J., Papathanasiou I.V., Fouly H.A., Kamau S.M., Valdez G.F.D., Adams K.A. (2018). Quality of life of nursing students from nine countries: A cross-sectional study. Nurse Educ. Today.

[52] Ramos-Dias J.C., Libardi M.C., Zillo C.M., Igarashi M.H., Senger M.H. (2010). Qualidade de vida em cem alunos do curso de Medicina de Sorocaba-PUC/SP. Rev. Bras. De Educ. Médica.

[53] Lins L., Carvalho F.M., Menezes M.S., Porto-Silva L., Damasceno H. (2016). Health-related quality of life of medical students in a Brazilian student loan programme. Perspect. Med. Educ..

[54] Torres G.C.S., Paragas E.D. (2019). Social determinants associated with the quality of life of baccalaureate nursing students: A cross-sectional study. Nurs. Forum.

[55] Meira T.M., Paiva S.M., Antelo O.M., Guimarães L.K., Bastos S.Q., Tanaka O.M. (2020). Perceived stress and quality of life among graduate dental faculty. J. Dent. Educ..

[56] Moutinho I.L.D., Lucchetti A.L.G., da Ezequiel O.S., Lucchetti G. (2019). Mental health and quality of life of Brazilian medical students: Incidence, prevalence, and associated factors within two years of follow-up. Psychiatry Res..

[57] Blázquez Abellán G., López-Torres Hidalgo J.D., Rabanales Sotos J., López-Torres López J., Val Jiménez C.L. (2016). Healthy eating and self-perception of health. Aten. Primaria.

[58] Govindaraju T., Sahle B.W., McCaffrey T.A., McNeil J.J., Owen A.J. (2018). Dietary Patterns and Quality of Life in Older Adults: A Systematic Review. Nutrients.

[59] Antonopoulou M., Mantzorou M., Serdari A., Bonotis K., Vasios G., Pavlidou E., Trifonos C., Vadikolias K., Petridis D., Giaginis C. (2020). Evaluating Mediterranean diet adherence in university student populations: Does this dietary pattern affect students’ academic performance and mental health?. Int. J. Health Plan. Manag..

[60] Ramón-Arbués E., Gea-Caballero V., Granada-López J.M., Juárez-Vela R., Pellicer-García B., Antón-Solanas I. (2020). The Prevalence of Depression, Anxiety and Stress and Their Associated Factors in College Students. Int. J. Environ. Res. Public Health.

[61] Ge Y., Xin S., Luan D., Zou Z., Liu M., Bai X., Gao Q. (2019). Association of physical activity, sedentary time, and sleep duration on the health-related quality of life of college students in Northeast China. Health Qual. Life Outcomes.

[62] Tao K., Liu W., Xiong S., Ken L., Zeng N., Peng Q., Yan X., Wang J., Wu Y., Lei M. (2019). Associations between Self-Determined Motivation, Accelerometer-Determined Physical Activity, and Quality of Life in Chinese College Students. Int. J. Environ. Res. Public Health.

[63] Yarmohammadi S., Ghaffari M., Yarmohammadi H., Hosseini Koukamari P., Ramezankhani A. (2020). Relationship between Quality of Life and Body Image Perception in Iranian Medical Students: Structural Equation Modeling. Int. J. Prev. Med..

[64] Yun E.K., Lee H., Lee J.U., Park J.H., Noh Y.M., Song Y.G., Park J.H. (2019). Longitudinal Effects of Body Mass Index and Self-Esteem on Adjustment From Early to Late Adolescence: A Latent Growth Model. J. Nurs. Res..

[65] Nguyen D.T., Wright E.P., Dedding C., Pham T.T., Bunders J. (2019). Low Self-Esteem and Its Association With Anxiety, Depression, and Suicidal Ideation in Vietnamese Secondary School Students: A Cross-Sectional Study. Front. Psychiatry.

[66] van Tuijl L.A., Bennik E.C., Penninx B.W.J.H., Spinhoven P., de Jong P.J. (2020). Predictive value of implicit and explicit self-esteem for the recurrence of depression and anxiety disorders: A 3-year follow-up study. J. Abnorm. Psychol..

[67] Sarwar S., Aleem A., Nadeem M.A. (2019). Health Related Quality of Life (HRQOL) and its correlation with academic performance of medical students. Pak. J. Med. Sci..

[68] Chattu V.K., Sahu P.K., Seedial N., Seecharan G., Seepersad A., Seunarine M., Sieunarine S., Seymour K., Simboo S., Singh A. (2020). An Exploratory Study of Quality of Life and Its Relationship with Academic Performance among Students in Medical and other Health Professions. Med. Sci..

[69] Keating X.D., Castelli D., Ayers S.F. (2013). Association of weekly strength exercise frequency and academic performance among students at a large university in the United States. J. Strength Cond. Res..

[70] Álvarez-Bueno C., Pesce C., Cavero-Redondo I., Sánchez-López M., Garrido-Miguel M., Martínez-Vizcaíno V. (2017). Academic Achievement and Physical Activity: A Meta-analysis. Pediatrics.

[71] Watson A., Timperio A., Brown H., Best K., Hesketh K.D. (2017). Effect of classroom-based physical activity interventions on academic and physical activity outcomes: A systematic review and meta-analysis. Int. J. Behav. Nutr. Phys. Act..

[72] Hershner S.D., Chervin R.D. (2014). Causes and consequences of sleepiness among college students. Nat. Sci. Sleep.

[73] Wang F., Bíró É. (2021). Determinants of sleep quality in college students: A literature review. Explore.

[74] Alotaibi A.D., Alosaimi F.M., Alajlan A.A., Bin Abdulrahman K.A. (2020). The relationship between sleep quality, stress, and academic performance among medical students. J. Fam. Community Med..

[75] Bolin D.J. (2019). Sleep Deprivation and Its Contribution to Mood and Performance Deterioration in College Athletes. Curr. Sports Med. Rep..

[76] Short M.A., Booth S.A., Omar O., Ostlundh L., Arora T. (2020). The relationship between sleep duration and mood in adolescents: A systematic review and meta-analysis. Sleep Med. Rev..

[77] Al Shammari M.A., Al Amer N.A., Al Mulhim S.N., Al Mohammedsaleh H.N., AlOmar R.S. (2020). The quality of sleep and daytime sleepiness and their association with academic achievement of medical students in the eastern province of Saudi Arabia. J. Fam. Community Med..

[78] El Hangouche A.J., Jniene A., Aboudrar S., Errguig L., Rkain H., Cherti M., Dakka T. (2018). Relationship between poor quality sleep, excessive daytime sleepiness and low academic performance in medical students. Adv. Med. Educ. Pract..

[79] Villalbí J.R., Bosque-Prous M. (2020). Policies to prevent the harm caused by alcohol: Priorities for Spain. Rev. Esp. De Salud Publica.

[80] Villalb J.R., Suelves J.M., Martínez C., Valverde A., Cabezas C., Fernández E. (2019). Smoking control in Spain: Current situation and priorities. Rev. Esp. De Salud Publica.

[81] Strack J., Esteves F. (2015). Exams? Why worry? Interpreting anxiety as facilitative and stress appraisals. Anxiety Stress Coping.

[82] Ringeisen T., Lichtenfeld S., Becker S., Minkley N. (2019). Stress experience and performance during an oral exam: The role of self-efficacy, threat appraisals, anxiety, and cortisol. Anxiety Stress Coping.

